# Recommendations from a Canadian Delphi consensus study on best practice for optimal referral and appropriate management of severe asthma

**DOI:** 10.1186/s13223-023-00767-6

**Published:** 2023-02-17

**Authors:** K. Godbout, M. Bhutani, L. Connors, C. K. N. Chan, C. Connors, D. Dorscheid, G. Dyck, V. Foran, A. G. Kaplan, J. Reynolds, S. Waserman

**Affiliations:** 1 Quebec Heart and Lung Institute, Laval University, Quebec City, Canada; 2 Department of Medicine, Division of Pulmonary Medicine, University of Alberta, Western Canada, Edmonton, AB Canada; 3 Department of Medicine, Dalhousie University, Halifax, NS Canada; 4 Faculty of Medicine, University of Toronto, Toronto, ON Canada; 5 Canadian Network for Respiratory Care, Bolton, Canada; 6 Centre for Heart Lung Innovation, St. Paul’s Hospital, Vancouver, BC V6Z 1Y6 Canada; 7 Clearspring Medical Clinic, Steinbach, MB Canada; 8 Canadian Anesthesiologists’ Society, Asthma Canada, Toronto, ON M4S 2Z2 Canada; 9 Family Physician Airways Group of Canada, Respiratory Effectiveness Group, Department of Family and Community Medicine, University of Toronto, Toronto, L4G 1N2 Canada; 10 Interim CEO, Asthma Canada, Toronto, ON M4S 2Z2 Canada; 11 Department of Medicine, Clinical Immunology and Allergy, McMaster University, Hamilton, ON Canada

**Keywords:** Asthma, Consensus development, Biologics, Consultation and referral, Primary care, Health care, Canada

## Abstract

**Background:**

In Canada, severe asthma affects an estimated 5–10% of people with asthma and is associated with frequent exacerbations, poor symptom control and significant morbidity from the disease itself, as well as the high dose inhaled, and systemic steroids used to treat it. Significant heterogeneity exists in service structure and patient access to severe asthma care, including access to biologic treatments. There appears to be over-reliance on short-acting beta agonists and frequent oral corticosteroid use, two indicators of uncontrolled asthma which can indicate undiagnosed or suboptimally treated severe asthma. The objective of this modified Delphi consensus project was to define standards of care for severe asthma in Canada, in areas where the evidence is lacking through patient and healthcare professional consensus, to complement forthcoming guidelines.

**Methods:**

The steering group of asthma experts identified 43 statements formed from eight key themes. An online 4-point Likert scale questionnaire was sent to healthcare professionals working in asthma across Canada to assess agreement (consensus) with these statements. Consensus was defined as high if ≥ 75% and very high if ≥ 90% of respondents agreed with a statement.

**Results:**

A total of 150 responses were received from HCPs including certified respiratory educators, respirologists, allergists, general practitioners/family physicians, nurses, pharmacists, and respiratory therapists. Consensus amongst respondents was very high in 37 (86%) statements, high in 4 (9%) statements and was not achieved in 2 (5%) statements. Based on the consensus scores, ten key recommendations were proposed. These focus on referrals from primary and secondary care, accessing specialist asthma services, homecare provision for severe asthma patients and outcome measures.

**Conclusions:**

Implementation of these recommendations across the severe asthma care pathway in Canada has the potential to improve outcomes for patients through earlier detection of undiagnosed severe asthma, reduction in time to severe asthma diagnosis, and initiation of advanced phenotype specific therapies.

## Background

Severe asthma (SA) is asthma that remains uncontrolled despite adherence with maximal inhaled therapy and treatment of contributory factors, or that worsens when high dose treatment is decreased [1]. In Canada, SA affects an estimated 5–10% of people with asthma [2] but is responsible for approximately 50% of all direct asthma-related costs [3]. SA represents a significant burden to the patient, as symptoms frequently interfere with day-to-day living, sleeping, and physical activity. In addition, patients experience frightening and unpredictable exacerbations/attacks [1]. Oral corticosteroids (OCS) are commonly used to manage exacerbations and gain control of symptoms but long-term use is associated with physical side effects including weight gain, development of cataracts, osteoporosis, hypertension and adrenal suppression, and psychological side effects such as anxiety and depression [1, 4]. Short-acting-β2-agonists (SABAs) are also used for symptom relief, but overuse is associated with increased risks of exacerbation and mortality [5]. Canadian data from two provincial datasets were analyzed as part of the global SABA in Asthma (SABINA) study. Results show that SABA overuse was substantial across both provinces (Nova Scotia: 39.4%; Alberta: 28.0%), and that the annual rate of asthma exacerbations was higher in patients with SABA overuse than in those without [6].

For these reasons, overuse of SABA and OCS treatments should be avoided. In addition to the symptoms of SA, comorbidities including dyspeptic disorders, bone loss, osteoporosis, cataract, and chronic kidney disease are common and contribute significantly to the patient burden. Comorbidity management has been shown to account for more than half of the incremental medical costs of SA patients in Canada [7].

Across Canada, significant heterogeneity exists in patient access to care. Broadly, there appears to be an over-reliance on SABAs and regular OCS use, two indicators of uncontrolled asthma which can indicate undiagnosed or suboptimally treated SA. A study of potential SA patients in Ontario treated with high-dose inhaled corticosteroids/long-acting beta agonists (ICS/LABAs) found that on average these patients visited their primary care physicians more than seven times in a year, but only 9% were referred for specialist care [8]. This could in part be due to low awareness of newer treatment options for SA amongst (non-specialist) primary care practitioners [9].

SA requires systematic assessment and characterization. To enable this, timely access to diagnostics should be universal in Canada, but this is not always the case. Spirometry testing is a key component in the accurate diagnosis of asthma (including SA) and is considered the gold standard by the Canadian Thoracic Society (CTS), but the availability of spirometry services varies between provinces and even among health regions within each province. For example, the Outaouais region in Quebec has 1.27 labs per 100,000 population, while the Nord-du-Québec region has 21 labs per 100,000 population [10].

Access to biologic therapies is also variable in Canada due in part to different reimbursement mechanisms in force across provinces and territories. SA is driven by different biological processes which are characterized by biomarkers such as fractional exhaled nitric oxide (FeNO), and induced sputum and blood eosinophil count [2], availability of biomarker testing facilities can therefore impact the diagnostic process for SA. Once SA phenotype has been established, a targeted biologic therapy should be used. To meet the needs of different SA phenotypes, a range of treatment options should be available to ensure equity of care.

The objective of this work is to build the first Canadian stakeholder consensus for diagnosis, appropriate referral, and treatment of SA. It is hoped that consensus around these important factors will help contribute to an improvement in the care delivered and ultimately, outcomes achieved for these patients.

## Methods

The Steering Group (an expert steering group of clinicians, authors cited in this work) met in 2022 to review the current landscape and systematically identify key topics in the SA care pathway through discussion of existing guidance and practice in Canada.

The eight key topics agreed by the Steering Group were:

1. Patient criteria for referral to a specialist
2. Role of the referring physician
3. Role of the receiving specialist
4. Initiation of advanced and other therapies
5. Access and capacity
6. Role of allied health care professionals in supporting severe asthma care and education
7. Performance measures
8. Patient empowerment

These topics were discussed in detail with the support of an independent Delphi facilitator (Triducive Partners Ltd.). The discussion culminated in the creation of 43 consensus statements for testing across a wider audience of clinicians involved in SA care in Canada. These statements were then used to develop a Likert questionnaire, which was sent out to HCPs (including pulmonologists, allergists, family doctors, nurses, pharmacists, certified respiratory educators, and respiratory therapists) identified by the expert steering group as working in relevant SA care services in Canada.

Respondents were offered a 4-point scale to rate their agreement with each statement, ranging across ‘strongly disagree’, ‘tend to disagree’, ‘tend to agree’ and ‘strongly agree’. Completed questionnaires were collated and the individual scores for each statement analyzed to produce an arithmetic agreement score for each.

The responses to consensus statements were analyzed in line with Delphi methodology [11]. It was agreed by the authors that a minimum of 100 responses would be appropriate.

The PRECISION Canada National Working Group predefined agreement for consensus at 75%, a widely accepted threshold [12]. Consensus was defined as ‘high’ at ≥ 75% and ‘very high’ at ≥ 90%. The final number of responses included in this analysis is 150.

## Results

**Fig. 1:**
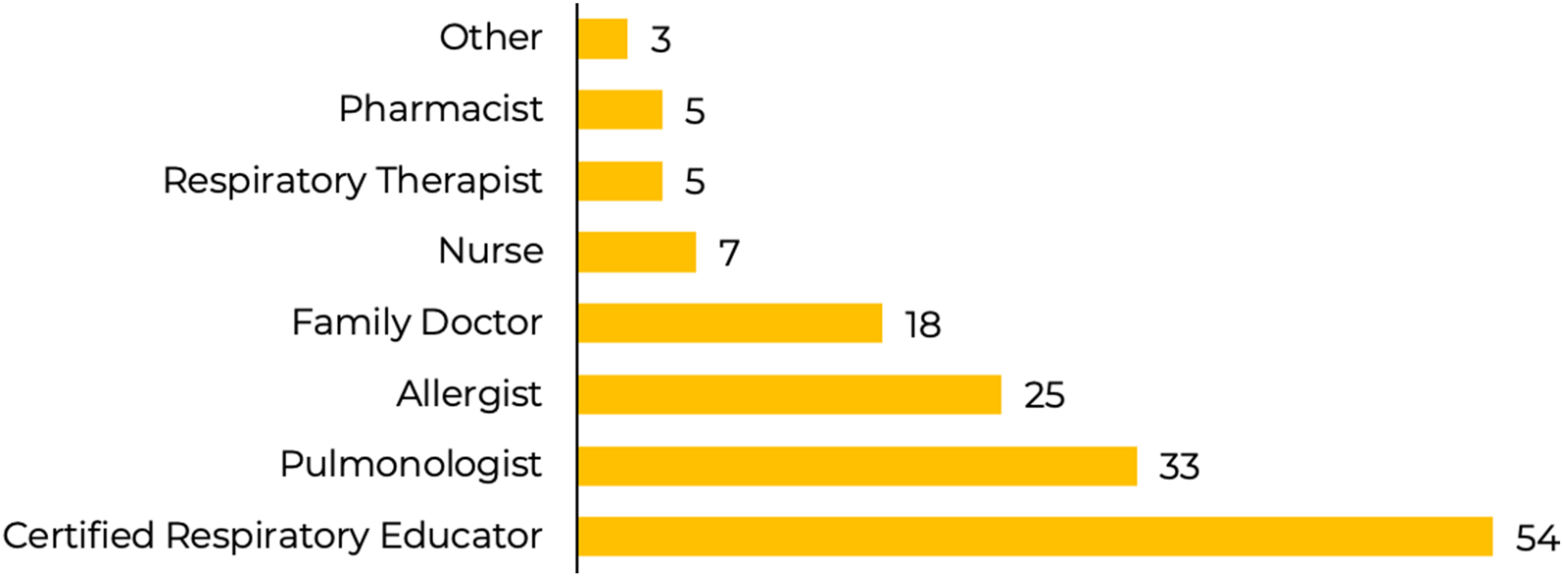
Respondents by role*.*Role of ‘Other’ includes healthcare managers and pediatricians, these roles have not been included individually in order to preserve anonymity, the role of ‘Nurse’ includes nurse practitioner and nurse

Completed questionnaires from 150 responders were analyzed to define the total level of agreement with each of the 43 statements. All respondents were professionals involved in the management of people with severe asthma, as shown in Fig. 1.

**Fig. 2:**
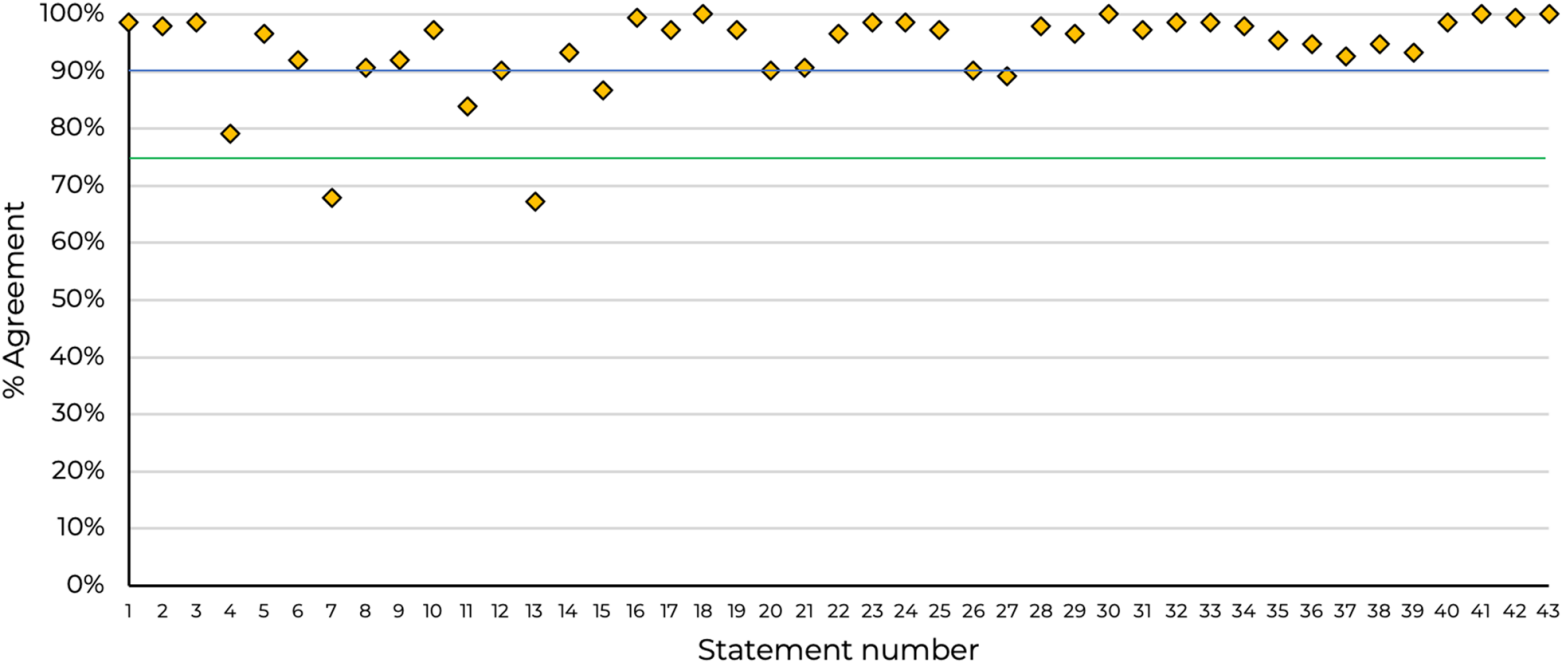
Overall consensus agreement levels by statement. Green horizontal line represents the 75% threshold for consensus agreement and the blue line indicates the threshold for very high consensus (90%)

**Fig. 3:**
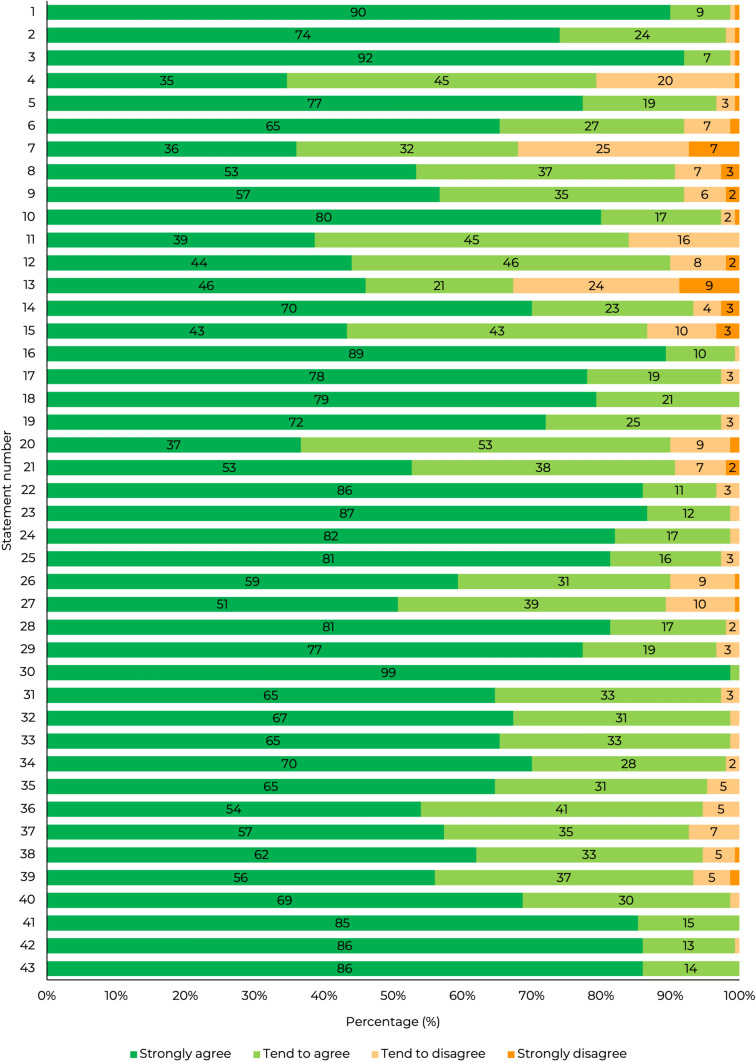
Agreement category of responses by statement. For clarity, labels for categories with ≤ 1% response have been removed

Consensus was very high (≥ 90%) in 37 (86%) statements, high (≥ 75 & ≤ 89%) in 4 (9%) statements and was not achieved (< 75%) in 2 (5%) of statements. Overall, forty-one statements achieved consensus (Fig. 2), responses according to topic are shown in Tables 1, 2, 3, 4, 5, 6, 7, 8. Percentage response to each statement by category of response is included in the Appendix (Fig. 3).

**Table 1:** Patient criteria for referral to a specialist (1–6)

No.	Statement	Agreement (%)
1	Patients who have used 2 or more courses of OCS and/or is using maintenance OCS therapy over the past 12 months despite adherence to high dose ICS/LABA therapy should be referred to a specialist	99
2	Patients who have had 1 or more emergency attendances /unscheduled visits due to asthma over the past 12 months despite adherence to high dose ICS/LABA therapy should be referred to a specialist	98
3	Patients who have ever been intubated or admitted to an ICU (intensive care unit) or high dependency unit despite adherence to high dose ICS/LABA therapy due to their asthma should be referred to a specialist	99
4	Patients who have used 3 or more SABA (short-acting beta2-agonist) inhalers in the past 12 months despite adherence to ICS therapy should be referred to a specialist	79
5	Patients with asthma who remain uncontrolled despite adherence to high dose ICS/LABA should be referred to a specialist	97
6	Health systems (including GPs, respiratory therapists, pharmacists and other healthcare professionals) across Canada should proactively case-find patients who meet criteria and flag for referral to a specialist OR severe asthma service	92

**Table 2:** Role of the referring physician (7–10)

No.	Statement	Agreement (%)
7	A positive diagnosis of severe asthma, confirmed by spirometry, should be achieved prior to any referral	68
8	All background steps (including a review of symptom control, treatments step, adherence, inhaler technique, co-morbidities, and risk factors) should be undertaken by the referrer prior to referral	91
9	Patient education resources should be accessed/provided (where available) by the referring physician prior to referral (including a discussion on co-morbidities, adherence, inhaler technique, smoking cessation, and lifestyle advice)	92
10	All documentation and the reason for referral should accompany the patient and be provided to the referrer with the referral to allow proper triage	97

**Table 3:** Role of the receiving specialist (11–16)

No.	Statement	Agreement (%)
11	Patients with diagnosed severe asthma need timely access (within 4 weeks) to a specialist	84
12	Patients with diagnosed severe asthma need timely access (within 8 weeks) to a specialist	90
13	Patients with diagnosed severe asthma need timely access (within 12 weeks) to a specialist	67
14	The receiving specialist should provide feedback to the referrer that the patient’s referral has been received and the anticipated date for that patient to be seen	93
15	The receiving specialist should be the owner of the onward management plan for the severe asthma patient	87
16	Ongoing communication between the receiving and referring physician improves outcomes for patients living with severe asthma	99

**Table 4:** Initiation of advanced and other therapies (17–21)

No.	Statement	Agreement (%)
17	Access to biologic therapies for patient (when indicated) is fundamental to improving outcomes in severe asthma in Canada	97
18	The choice of biologic therapies should be driven by phenotyping, which includes clinical history (e.g., triggers, age of onset), comorbidities, biomarkers and spirometry	100
19	Future Canadian severe asthma guidelines should include pragmatic and practical guidance on the initiation and choice of biologic therapies for severe asthma	97
20	Once a patient has been approved for an advanced therapy, initiation of treatment should not be delayed by more than 2 weeks	90
21	Once a patient has been approved for an advanced therapy, initiation of treatment should not be delayed by more than 4 weeks	91

**Table 5:** Access and capacity (22–27)

No.	Statement	Agreement (%)
22	Access to primary care is fundamental to improving outcomes in severe asthma in Canada	97
23	Access to specialist care is fundamental to improving outcomes in severe asthma in Canada	99
24	There must be equality of access to appropriate severe asthma care irrespective of geographic location	99
25	Access to diagnostic tools (including spirometry, lung function test etc.) within 4 weeks is fundamental to improving outcomes in severe asthma in Canada	97
26	Access to educators (e.g., nurse, CRE) within 2 weeks prior to referral is fundamental to improving outcomes in severe asthma in Canada	90
27	Access to virtual (digital and/or tele-health) models of care should be promoted in Canada to improve the care of patients with severe asthma	89

**Table 6:** Role of allied health care professionals in supporting severe asthma care and education (28–30)

No.	Statement	Agreement (%)
28	Allied healthcare professionals (nurses, educators, respiratory therapists, pharmacist, etc.) have a critical role to fill in the ongoing management of severe asthma patients	98
29	Pharmacy (either community- or hospital-based) has an important role to play in helping to identify asthma patients who have an over-reliance on SABA and/or OCS use or are non-adherent	97
30	All patients with asthma need to be educated about their asthma	100

**Table 7:** Performance measures (31–38)

No.	Statement	Agreement (%)
31	National Pan-Canadian data collection about severe asthma needs to be both established and sustained	97
32	Establishing a benchmark and capturing data on emergency room use would help improve healthcare planning, delivery, and outcomes of severe asthma	99
33	Establishing a benchmark and capturing data on healthcare utilization (hospitalization) would help improve healthcare planning, delivery, and outcomes of severe asthma	99
34	Establishing a benchmark and capturing data on steroid (OCS) use would help improve planning, delivery, and outcomes of severe asthma	98
35	Establishing a benchmark and capturing data on SABA use would help improve planning, delivery, and outcomes of severe asthma	95
36	Patient-centered performance measures should be established and tracked in Canada	95
37	Results of performance measures should be published in Canada	93
38	National guidelines and statements relating to severe asthma care should be published as a single authoritative source	99

**Table 8:** Patient empowerment (39–43)

No.	Statement	Agreement (%)
39	Patients living with the symptoms of uncontrolled asthma should expect and demand more from their asthma care in Canada	93
40	A change in belief of asthma control would be beneficial and eliminate the normalization of over-use of SABA and/or OCS medicines	99
41	Severe asthma patients should be empowered to co-manage their condition in partnership with their healthcare provider	100
42	Patients living with uncontrolled asthma should receive a timely, formal diagnosis and quality care by an expert team	99
43	A common and clear understanding of asthma control would be beneficial for both patients and HCPs to help eliminate the normalization of asthma symptoms and over-use of SABA and/or OCS medicines	100

## Discussion

In Canada there is significant variation in how patients with SA are diagnosed and cared for. There are multiple patient pathways reflecting local expertise and service and system structures. In addition, there are a limited number of specialist SA centers across Canada [13] and capacity within these centers is therefore finite. The geographical distribution of specialist centers may mean that some patients have to travel considerable distance for specialist severe asthma care. Most patients report that they receive their asthma care from a primary care physician and 53% indicated that long wait times to see specialist prevents them from receiving asthma care, support, and treatment required [14].

Where SA is suspected, the primary care provider may refer the patient to an allergist or respirologist within the local secondary care service. In some situations, the specialist may need to refer the patient on to a severe asthma center.

The first step in identification of SA is identifying those individuals with uncontrolled asthma. Interrogation of family physician prescribing data or pharmacy dispensing data may provide insight into OCS and SABA use and identify those patients who may have poor adherence. Uncontrolled patients should be reviewed by their family physician and appropriate optimization and patient education provided. If the patient remains uncontrolled despite good adherence to maximal inhaled therapy (through correction of inhaler technique, management of comorbidities etc.), then respondents agree strongly that a referral to an asthma specialist should be made (Statements 1- 5). Pharmacists may be able to support in identifying those individuals with either a pattern of overuse of SABA or OCS, or who do not collect renewals of their maintenance medications at the expected frequency (suggesting poor adherence).

It is interesting that the response to statement 4, while still above the consensus agreement threshold, scored significantly lower than statements 1, 2, 3 and 5. This could be due to the wording of the statement, which uses the term ‘ICS therapy’ rather than the ‘high dose ICS/LABA’ used in other statements, and should have been revised for more clarity. Another reason may be that the overuse of SABA inhalers is common in Canada despite the clear relationship between SABA overuse and worsening asthma control, increased risk of exacerbations, and mortality [5]. To address this pattern of behavior, a strong message is needed within the asthma community that overuse of SABA therapy should be challenged, and patients assessed and referred promptly as required.

There was a clear lack of agreement regarding the use of spirometry in SA (Statement 7; 68%). This raises some important questions: is this belief due to a disagreement with the use of spirometry or is it due to the reality of limited access to spirometry services in some areas? While spirometry is the gold standard for asthma diagnosis, there are situations where it may be inconclusive, and with the diagnosis of SA being based on medication use and control of symptoms, some may feel that spirometry is not required for a referral. Sub-analysis of this statement by role shows the lowest levels of agreement were amongst respirologists and family physicians (57% and 60%, respectively), while the highest were amongst nurses and CREs (86% and 76%, respectively). It is reasonable to assume that in areas with limited access to spirometry, HCPs are not prepared to wait, particularly when patients may require access to biologic therapies. In practical terms, where spirometry is not available in a timely manner, a lack of asthma control which persists after treatment and adherence optimization should prompt a referral.

There was clear agreement that the patient should be reviewed (Statement 8; 91%) and provided with appropriate educational support (Statement 9; 92%) prior to any referral. This is a key step that would help poorly controlled patients gain control of their asthma and prevent unnecessary referrals, thereby helping to minimize capacity needs in specialist clinics.

Statements 11–13 were intended to gauge responder opinion on the optimum time for patient to be seen by a specialist after referral. Statement 13 was supported by many respondents (67% agreement) but did not achieve consensus agreement, on further analysis there was a clear variation in response by region with Manitoba, Nova Scotia, and Newfoundland and Labrador having the highest agreement levels of agreement (88–100%), and Quebec and British Columbia, the lowest levels (48–50%). Further work is needed to understand these differences in opinion.

Overall, the response to these data suggests that 4–8 weeks is optimal, and that 12 weeks is less acceptable. This is in line with the findings of a recent consensus in the UK and is not considered a failure to achieve consensus [15].

Respondents strongly agreed that receiving specialists should notify the referrer that the referral has been received and an indication of when the patient can be seen (assuming the referral is accepted). This is already specified in the guidelines of most provincial medical regulatory authorities [16–18] and should be standard practice, although anecdotal experience of the authors suggests that this is variable in practice. The circle of care for the patient is dependent on clear communication between HCPs, but unfortunately there are gaps. In 2019, 14.5% of Canadians aged ≥ 12 years did not have access to a family physician, and this was highest in Quebec (21.5%), Saskatchewan (17.2%), and British Columbia (17.7%) [19]. As a consequence, these patients may rely on episodes of care from a hospital, emergency room, or walk in clinic, a model of care that can be described as fractured at best.

All statements reported in Table 4 achieved consensus agreement with 3/5 achieving over 95% agreement amongst responders.

SA often requires treatment with a biologic medication. Asthma (and by extension SA) is increasingly understood to be an umbrella term for several diseases with distinct inflammatory mechanisms (endotypes) and variable clinical presentations (phenotypes). Characterization of patient endotype allows targeted use of biologic therapies. There are a range of biologic treatments approved for use, and these differ in their molecular targets and the subsequent impact on inflammatory pathways [20]. Access to a range of biologic therapies is therefore essential to managing SA, but there is considerable variation across provinces [9].

Respondents strongly agreed that future Canadian guidelines should provide pragmatic and practical guidance regarding the initiation and choice of biologic therapies (Statement 19, 97%). It is possible that such guidelines could support a consistent offering of advanced therapies across provinces and territories, a ‘minimum offer’ that would provide equity of access for all patients.

There was strong agreement that once approved for a biologic therapy, treatment should be initiated within two to four weeks (90% and 91%, respectively).

All statements reported in Table 5 achieved very high consensus agreement. The overarching theme of these results is that a range of services should be in place and available in a timely manner to provide the infrastructure to deliver optimal care for patients with SA.

Respondents agreed that access to diagnostic tools within 4 weeks is fundamental to improving SA outcomes in Canada (Statement 25, 97%), suggesting that HCPs recognize the value of spirometry, lung function tests etc., despite variations in access. This also suggests that the lack of consensus for statement 7 is more likely related to access to services rather than a lack of belief in the value of these diagnostic methods.

Virtual care has rapidly been adopted in many countries in response to the COVID-19 pandemic. The term covers a range of communication methods including video calls, telephone, email, and even remote monitoring of personal diagnostic devices such as blood glucose monitors. Canada is a large and relatively sparsely populated country, and access to healthcare is a geographical challenge for some. In this situation, virtual methods are extremely valuable, but not all people prefer (or have access to) virtual care. In addition to this, some healthcare activities require in-person attendance (e.g., for examination or diagnostic procedures), so virtual care methods should be offered but appropriate to the individual patient.

Statement 26 had a slightly lower agreement level than statements 22–25 (90%), and this may be due to the wording of the statement, and whilst many agree that access to educators is important in SA care, they may not all agree that access within 2 weeks is ‘fundamental’. The authors suggest that referrals for suspected SA should be made even if access to educators is limited, as there is still opportunity for patient education to be delivered while the referral is in progress.

Almost all respondents (regardless of role) agreed that allied healthcare professionals are critical to the ongoing management of severe asthma patients.

All (100%) of respondents recognized the need for patients to be educated about their asthma. In Canada, certified respiratory educators (and certified asthma educators) are in place to deliver consistent and high-quality education to patients and HCPs. Certified respiratory educators (CRE) represent a range of healthcare roles, including pharmacists, nurses, occupational therapists, and respiratory therapists, and there are currently around 1,500 CREs and 375 CAEs in Canada [21]. In 2021, Asthma Canada report that 19% of patients had difficulty in accessing an educator most of the time and a further 25% had difficulty some of the time. This suggests a need for greater provision of education from dedicated practitioners, indeed, this was a key policy recommendation by Asthma Canada in 2019 [14, 22].

Respondents also strongly agreed that pharmacy services can play an important role and helping to identify uncontrolled asthma patients for appropriate follow up and referral.

There is a lack of national publicly available asthma specific outcomes data for Canada. Respondents strongly agreed that there is a need to establish a national data collection (S31, 97%), which can be used to develop benchmarks (S32-35, 95–99%) and performance measures (S36, 95%) for asthma care. Publication of patient-centered measures (S37, 93%) would allow patients to make more informed decisions about their healthcare.

Patient reported outcomes measures (PROMs) are growing in importance and are often defined as secondary endpoints in Phase 3 clinical studies [23]. PROMs can be collected using an agreed, validated tool (e.g., Asthma Quality of Life Questionnaire (AQLQ), Asthma Control Questionnaire (ACQ), and the Asthma Symptoms Diary (ASD)), and this this approach should be embedded in routine clinical practice.

Guidelines should be published as a single authoritative source (S38, 99%) to provide a consistent and evidence-based approach for clinical practice. Such guidelines do require a concerted effort to develop and can quickly become obsolete if not updated regularly. In Canada, a survey of 234 HCPs involved in asthma care found that 77% reported sub-optimal knowledge of Global Initiative for Asthma (GINA) guidelines for adult asthma care, compared with 64% for the CTS guidelines (which were last updated in 2017, and therefore may be obsolete regarding newer treatments) [24]. A single source could be beneficial in providing a consistency of approach and increasing familiarity and understanding amongst HCPs.

Respondents clearly agreed that patients should expect to receive quality care for their severe asthma (S39; 93%, S42; 99%). To enable this, the normalization of SABA and OCS overuse amongst both patients and HCPs should be challenged (S40, 99%). The Asthma Canada Severe Asthma Patient Charter [25] sets out the key principles of patient expectations, patients should be aware of the expectations of care in severe asthma to drive improvement.

It is interesting to note that S39 achieved a slightly lower level of agreement (93%) than the other statements in this topic, this could be due to the use of the word ‘demand’ and the context that responses were from HCPs, not all of which may wish patients to make further demands. Future work could involve a patient specific survey to gauge level of agreement with the principles described here.

## Recommendations

Due to the very high levels of consensus for all but two of the statements, only one round of questionnaire was required. The results of the survey represent current opinions of the respondents and are not intended to contradict the established evidence base. Overdiagnosis of asthma is a recognized issue [26], therefore the recommendations below refer to the management of patients with a confirmed asthma diagnosis.

**Table:** 

Recommendations	
1	Primary care clinicians should pro-actively identify suspected severe asthma patients for optimization (including appropriate referral)
2	Pharmacy (either community- or hospital-based) should be utilized to help identify potential severe asthma patients
3	Asthma patients that have a history of overuse of SABA and/or repeat OCS therapies should be assessed for severe asthma
4	Access to diagnostic tools (including spirometry, lung function test etc.) within 4 weeks of request should be an expected standard within Canada
5	A consistent pathway for referral of suspected severe asthma patients should be in place across Canada with clearly defined criteria and acceptable waiting time
6	All patients should receive education about their asthma from an asthma educator
7	The choice of biologic therapy should be driven by disease phenotype, which is determined by clinical history, comorbidities, biomarkers and spirometry
8	Initiation of a biologic therapy should be within 2–4 weeks of approval
9	National Pan-Canadian data collection about severe asthma should be established
10	Patients should be empowered to work together with their HCPs, through shared decision-making tools, to manage their symptoms and control their asthma

## Strengths and limitations of this study

Strengths include:

- Expert opinion used to inform questionnaire
- Opinions of a representative number of HCPs in a variety of roles and locations across Canada were used to inform recommendations
- 150 responses gained across a range of HCP roles working in SA

Limitations include:

- Selection of respondents subject to bias as the survey was sent out to professional HCP networks by the working group
- The high levels of agreement achieved may have been an indication that the statements were constructed to be allow confirmation bias during interpretation, while this was not the intention, the authors acknowledge this possibility.
- Whilst patient experience was discussed and there was representation from Asthma Canada on the steering group, there was no survey of patient views
- The number of responses from Pharmacists, Respiratory Therapists, and Nurses were low (≤ 10), further work should seek to address this.
- The largest responder group by role was CRE (including CAE) with 54 responders, but CREs may also have other primary healthcare roles (i.e., pharmacist, respiratory nurse, etc.), we did not ask for clarification of role within this group, and this may have added additional insight.

## Conclusion

The Steering Group was able to form a strong set of recommendations based on the high levels of agreement achieved for most statements. It is hoped that adoption will improve detection of severe asthma, reduction in time to diagnosis and initiation of advanced phenotype-specific therapies.

## Data Availability

The datasets used and/or analyzed during the current study are available from the corresponding author on reasonable request.

## Appendix

See Fig. 3

## Abbreviations

ACQ: Asthma Control Questionnaire
AQLQ: Asthma Quality of Life Questionnaire
ASD: Asthma Symptoms Diary
CAE: Certified asthma educator
CRE: Certified respiratory educator
FeNO: Fractional exhaled nitric oxide
HCP: Healthcare professional
ICS: Inhaled corticosteroid
LABA: Long-Acting Beta2-Agonist
OCS: Oral corticosteroid
PROM: Patient reported outcomes measure
SABA: Short-Acting Beta2-Agonist
